# Supplementary material for: Improving organic photovoltaic cells by forcing electrode work function well beyond onset of Ohmic transition
Source: Nat Commun. 2021 Apr 14;12:2250. doi: 10.1038/s41467-021-22358-y (PMC8047006; doi:10.1038/s41467-021-22358-y)
Supplement: Supplementary file 1 — Supplementary Information [file 41467_2021_22358_MOESM1_ESM.pdf]

Improving organic photovoltaic cells by forcing electrode work function well beyond onset of Ohmic transition

Chao ZHAO,[1,3] Cindy G. TANG,[1] Zong-Long SEAH,[1] Qi-Mian KOH,[2] Lay-Lay CHUA,[1,2] Rui-Qi PNG,[1] Peter K.H. HO[1]*

[1] Department of Physics, National University of Singapore, Lower Kent Ridge Road, S117550, Singapore

[2] Department of Chemistry, National University of Singapore, Lower Kent Ridge Road, S117552, Singapore

[3] Present address: State Key Laboratory for Mechanical Behavior of Materials, Xi'an Jiaotong University, Xi'an 710049, Shaanxi, People's Republic of China

* Correspondence to:
P.K.H.H. (phyhop@nus.edu.sg)

Supplementary Information

Contents

1. Supplementary Figures 2

2. Supplementary Table 17

a PBDTTPD core: 4½-repeat-unit

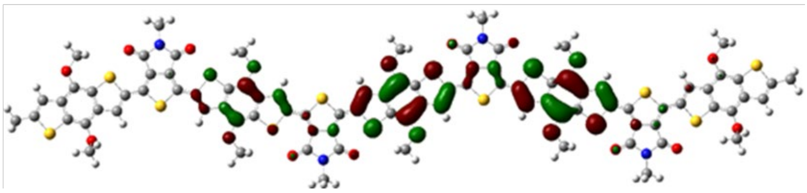

ℓ	E_{HOMO} (eV)	E_{HOMO-1} (eV)	E_{HOMO-2} (eV)	E_{HOMO-3} (eV)	E_{HOMO-4} (eV)
1½	−7.69	−7.87	--	--	--
2½	−6.16	−6.29	−6.49	--	--
3½	−6.15	−6.24	−6.33	−6.49	
4½	−6.16	−6.25	−6.34	−6.49	−6.54
5½	−6.15	−6.22	−6.30	−6.36	−6.50

b P3HT core: 12-repeat-unit

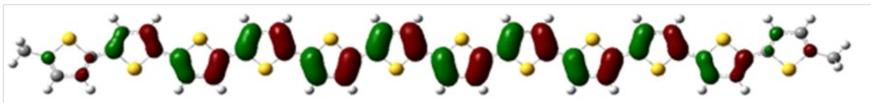

ℓ	E_{HOMO} (eV)	E_{HOMO-1} (eV)	E_{HOMO-2} (eV)	E_{HOMO-3} (eV)	E_{HOMO-4} (eV)
8	−6.36	−6.83	−7.29	−7.76	
10	−6.27	−6.64	−7.01	−7.39	−7.76
12	−6.21	−6.52	−6.83	−7.14	−7.45
14	−6.16	−6.43	−6.69	−6.96	−7.23
16	−6.13	−6.36	−6.59	−6.83	−7.06

Supplementary Figure 1. Molecular-orbital wavefunctions and energies for gas-phase semiconductor cores. (a) PBDTTPD core, and **(b)** P3HT core. Methodology: Wavefunctions and energies were computed at DFT/CAM-B3LYP/6-311G after geometry optimization at PM3 in the planar conformation. No significant difference (< 0.01 eV) was found for twisted conformations with dihedral angles up to ±5°. Wavefunctions are shown for 4½-repeat-unit PBDTTPD core, and 12-repeat-unit P3HT core, at a surface isovalue of 0.02. The '½-unit' in PBDTTPD accounts for symmetric termination of both ends by BDT moiety. Inspection of E_{HOMO} and E_{HOMO-1} as a function of number of repeat units (ℓ) shows the hole localizes more strongly in PBDTTPD. This produces non-dispersive wavefunction energies for $\ell > 2½$. From experimental data, the mean transport-conjugation-length ($<\ell>$) is estimated to be 3½ units for PBDTTPD, and 12 units for P3HT.

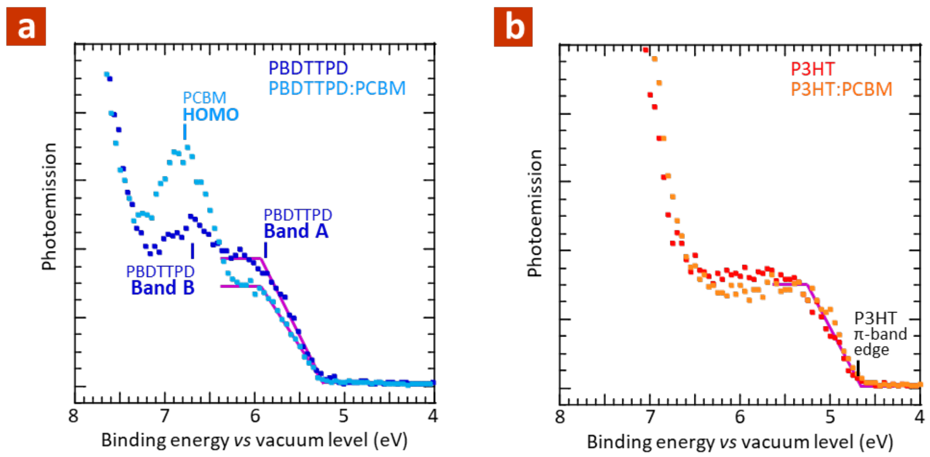

Supplementary Figure 2. Ultraviolet photoemission spectroscopy of valence band. (a) PBDTTPD and PBDTTPD:PCBM (1:1.5 w/w) films, and **(b)** P3HT and P3HT:PCBM (1:0.8 w/w). The photoelectron inelastic mean free path at these kinetic energies is *ca*. 0.6 nm. Bands A and B of PBDTTPD correspond to wavefunctions with primary nodal planes parallel and perpendicular to chain axis, respectively. Their energy split of 0.8 eV agrees with the computed difference of 1.1 eV by DFT/CAM-B3LYP/6-311G. The frontier π-band of P3HT is strongly dispersive, spanning several eV and producing a gradual stepped onset. Magenta '*z*' lines are guides-to-the-eye for the valence-band 'width' of the frontier region. The widths of PBDTTPD and PBDTTPD:PCBM films are identical, and larger than those of P3HT and P3HT:PCBM films. All films were measured consecutively in identical configuration with same He-I radiation intensity.

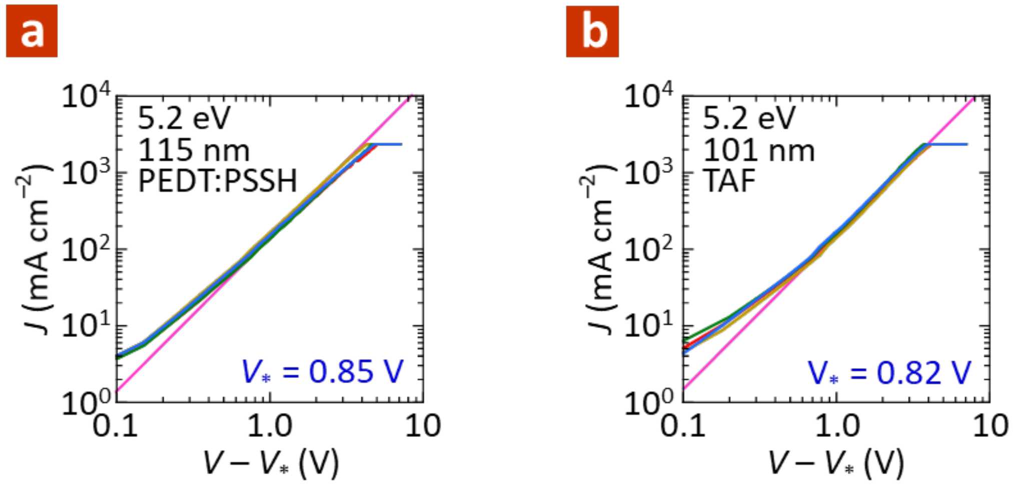

Supplementary Figure 3. ***JV*** **characteristics of hole-only PBDTTPD:PCBM diodes.** Diode structure: ITO/HIL/PBDTTPD:PCBM/Ag, where HIL is the hole injection layer: **(a)** PEDT:PSSH, and **(b)** TFOMe-CF_3SIS, both with ϕ of 5.2 eV. Typical data are shown for four diodes in the backward scan direction. Magenta line marks the ideal slope of 2.0 for Mott–Gurney behaviour: $J = \frac{9}{8}\varepsilon_r\varepsilon_o\mu_{eff}\frac{(V-V_*)^2}{d^3}$, where V_* is the apparent V_{bi}, evaluated from the linear-linear Mott–Gurney plot. The measured *JV* characteristics obey this equation over 1.5 decades of *J*. Deviation at low voltage is due to diffusion-current contribution; flat segment at high voltage is due to compliance limitation (2,300 mA cm^{-2}). PBDTTPD:PCBM film thickness is given in plot, ± 5 nm. Despite similar work functions, TFOMe-CF_3SIS provides better hole injection than PEDT:PSSH, as evidenced by lower V_* and more ideal Mott–Gurney index (see also Supplementary Fig. 10).

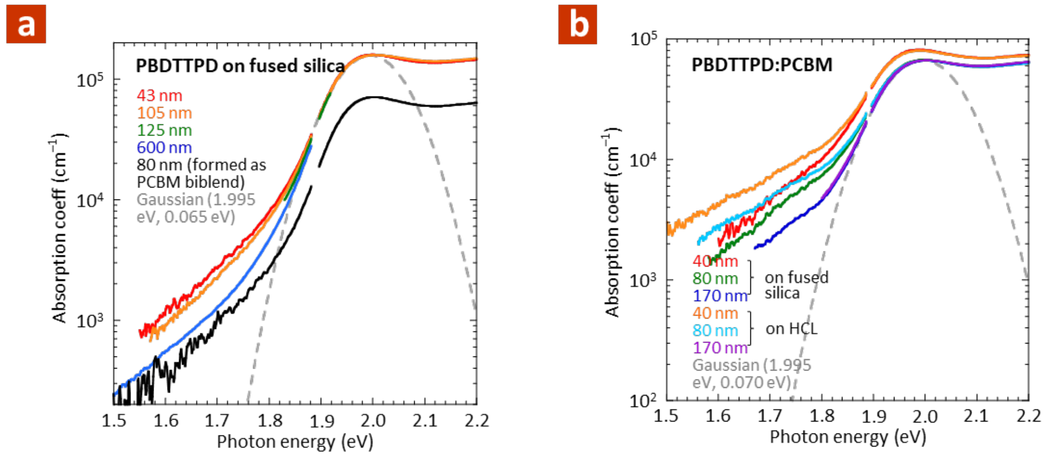

Supplementary Figure 4. Optical spectra of PBDTTPD and PBDTTPD:PCBM. (a) PBDTTPD films on fused silica, and **(b)** PBDTTPD:PCBM films on fused silica or HCL. Methodology: Index-matched UV–Vis spectroscopy. All films were spin-cast from chlorobenzene solution at room temperature, and measured without annealing. A refractive-index-matching fluid (F) was applied to eliminate reflection and thin-film-interference artefacts. For (a), we used the configuration: air/fused silica/PBDTTPD/F/fused silica/air, or air/fused silica/PBDTTPD/F/PBDTTPD/fused silica/air; for (b), air/fused silica/optional HCL/PBDTTPD:PCBM/F/fused silica/air, where HCL is a 15-nm-thick hole-doped TFB-CF_3SIS film with ϕ of 5.35 eV. For acquiring PBDTTPD spectra, F is decane (refractive index, 1.41); for PBDTTPD:PCBM spectra, ethylene glycol (1.43). Decane extracts PCBM from the 80-nm-thick biblend film in (a), initially formed as PBDTTPD:PCBM (1:1 w/w), to give the remaining PBDTTPD matrix. All spectra were collected in the nitrogen glovebox. Data processing: The constant reflection loss from both air interfaces was subtracted. Selected spectra were fitted to Gaussians—the mean energy and standard deviation (σ_{opt}) are given in sequence inside brackets (,). PBDTTPD films give good match out to 2.3σ. On the other hand, PBDTTPD:PCBM films exhibit strong sub-gap absorptions, attributed to PCBM→PBDTTPD and polaron transitions. The expected PCBM absorption at 1.76 eV, with absorption coefficient of 1.5 x 10^3 cm^{-1}, is submerged. Films on HCL additionally exhibit a polaron absorption due to hole doping of their interfaces. This manifests as apparent inverse thickness dependence. For both P3HT and P3HT:PCBM films, literature gives σ_{opt} to be *ca*. 0.05 eV. Reference: W.J.D. Beenken, F. Herrmann, M. Presselt, H. Hoppe, S. Shokhovets, G. Gobsch, E. Runge, Phys. Chem. Chem. Phys. 15 (2013) 16494.

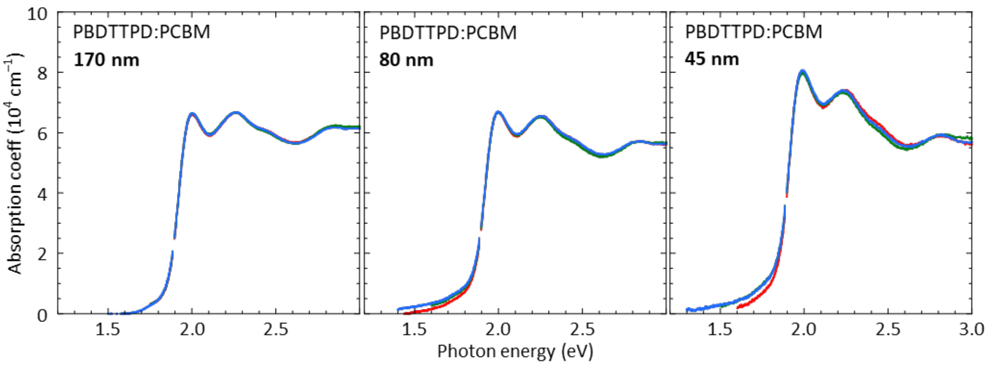

Supplementary Figure 5. Optical spectra of PBDTTPD:PCBM films on fused silica. Legend: red, without HCL; green, with hole-doped TFB-CF_3SIS; and blue, hole-doped mTFF-C_2F_5SIS, as HCL. Methodology: Anhydrous acetonitrile solutions of hole-doped TFB-CF_3SIS, or mTFF-C_2F_5SIS, were spin-cast in nitrogen glovebox onto oxygen-plasma-cleaned fused silica substrates to give 15-nm-thick films, with ϕ of 5.35 eV, and 5.75 eV, respectively. PBDTTPD:PCBM films with different thicknesses were then spin-cast from chlorobenzene at appropriate concentrations. Optical spectra were collected without annealing in the nitrogen glovebox. Refractive-index matching with ethylene glycol was used to suppress reflection and thin-film interference artefacts, using the configuration: air/fused silica/optional HCL/PAL/F/fused silica/air. Data processing: Small vertical rescaling was applied to superpose all spectra in the same dataset for shape comparison. The polaron absorption of the hole-doped PBDTTPD interface ($\lesssim$ 1.8 eV) on HCL films can be discerned in the thinner PBDTTPD:PCBM film less than or equal to 80-nm thick.

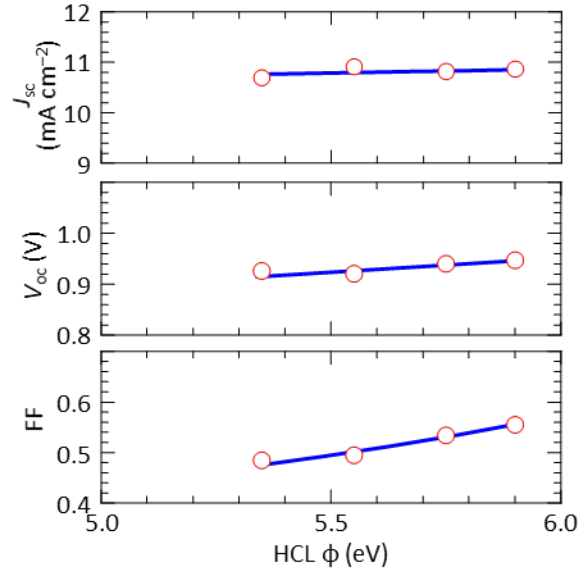

Supplementary Figure 6. Parameters for inverted PBDTTPD:PCBM solar cells with different top HCL work functions. Cell structure: ITO/10-nm ECL/100-nm PBDTTPD:PCBM (1:1.5 w/w)/10-nm HCL/Ag, where ECL is a spin-on film of self-compensated, electron-doped poly(fluorene–*alt*–benzothiadiazole) with ϕ of 3.3 eV, and HCL is a spin-on film of self-compensated, hole-doped TAF polymer with selected ϕ, deposited from acetonitrile solutions. Both PEDT:PSSH (aqueous solution) and TFOMe-CF_3SIS, with ϕ of 5.20 eV, could not be deposited onto the PAL due to non-wettability. Measurement conditions: AM1.5G (simulated) at 100 mW cm^{-2}. Data presentation: Symbol size is larger than standard error ($n \geq 4$). Blue lines are guides-to-the-eye.

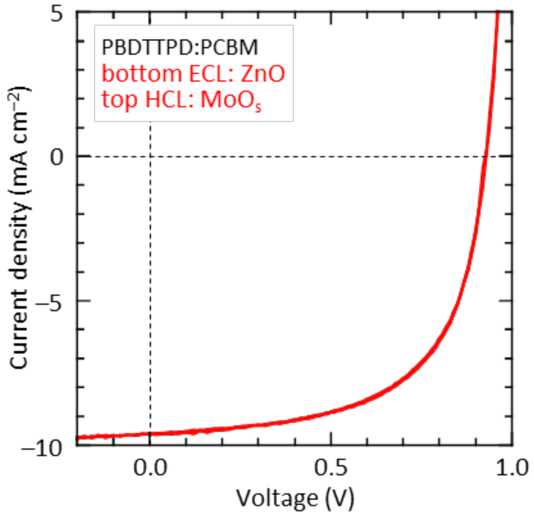

Supplementary Figure 7. Representative *JV* characteristic of PBDTTPD:PCBM solar cells with ZnO and MoO_3 charge collection layers. Cell structure: ITO/40-nm ZnO/100-nm PBDTTPD:PCBM (1:1.5 w/w)/7-nm MoO_3/Ag, where ZnO was deposited by sol–gel process and baked at 150°C for 10 min in air on hotplate to give the electron collection layer, and MoO_3 was deposited by evaporation to give the hole collection layer. Measurement conditions: AM1.5G (simulated) at 100 mW cm^{-2}. Typical parameters: V_{oc} = 0.93 V, J_{sc} = 9.7 mA cm^{-2}, FF = 0.60, PCE = 5.4%. Solar cells where MoO_3 is replaced by 10-nm-thick hole-doped mTFF-C_2F_5SIS, spin-cast from acetonitrile solution, give: V_{oc} = 0.97 V, J_{sc} = 7.7 mA cm^{-2}, FF = 0.66, PCE = 4.9%.

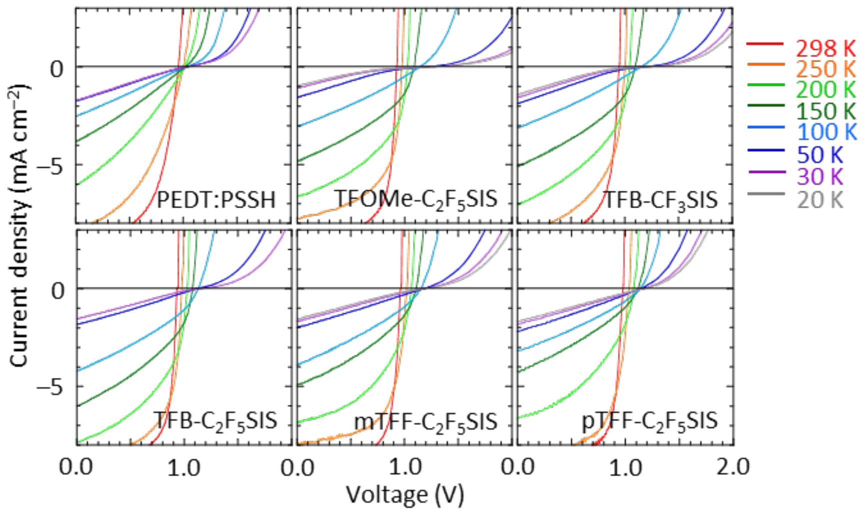

Supplementary Figure 8. Variable-temperature *JV* characteristics of PBDTTPD:PCBM solar cells. Cell structure: ITO/20-nm HCL/100-nm PBDTTPD:PCBM (1:1.5 w/w)/30-nm Ca/120-nm Al. Measurement conditions: AM1.5G (simulated) at 100 mW cm^{-2}. The HCL is given in the panel. Dark leakage correction was performed for the low-temperature *JV* curves wherever required.

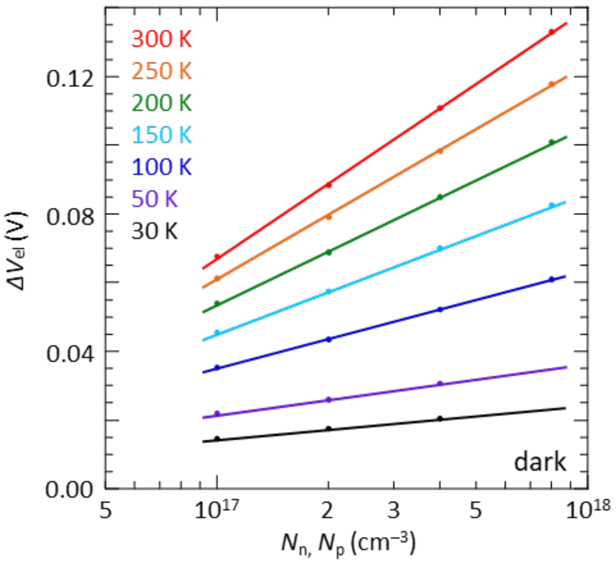

Supplementary Figure 9. Total electrostatic band-bending voltage loss as a function of majority carrier density at contacts. Methodology: Drift–diffusion modeling of electrostatic band-bending (ΔV_{el}) due to carrier diffusion. Symmetrical contacts are assumed in the dark and for flat-band condition. Identical majority carrier densities are also assumed at both electron and hole contacts, i.e. $N_n = N_p$. Other parameters: carrier mobilities, $\mu_p = \mu_n = 5 \times 10^{-4}$ cm^2 V^{-1} s^{-1}; film thickness, 100 nm. The functional dependence of ΔV_{el} on N_n and N_p is logarithmic in the range of interest. This ΔV_{el} partially counteracts the increase in V_{bi} with electrode ϕ, i.e. $V_{bi} = V_o - \Delta V_{el}$.

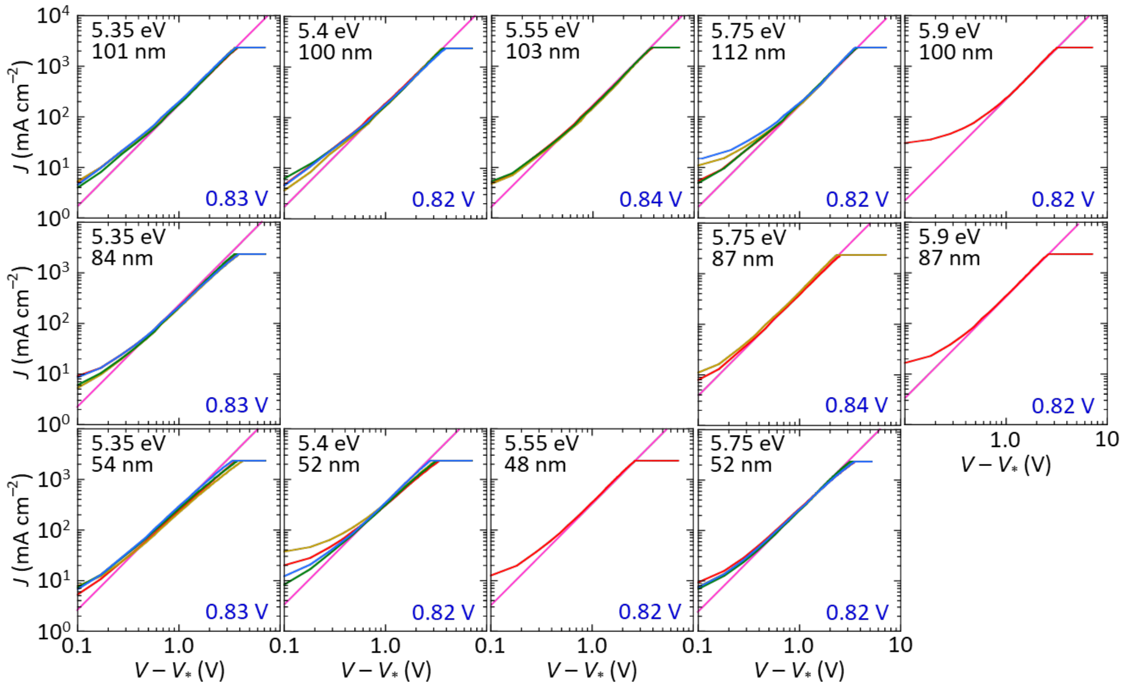

Supplementary Figure 10. *JV* characteristics of hole-only PBDTTPD:PCBM diodes in log–log plot. Device structure: ITO/TAF/PBDTTPD:PCBM/Ag, where TAF acts as hole injection layer. Representative data are shown for the backward sweep direction. TAF work function (± 0.05 eV) and PBDTTPD:PCBM film thickness (± 5 nm) are given at top left of each plot. Magenta line gives the ideal Mott–Gurney slope, i.e. $\frac{d\log J}{d\log(V-V_*)}$, of 2.0, with V_* given at bottom right of each plot. The measured *JV* characteristics generally obey the ideal Mott–Gurney law over 1–1.5 decades of current density. Deviation at low voltage is due to diffusion and leakage current contributions. The flat segment at high voltage is due to compliance limitation (2,300 mA cm^{-2}).

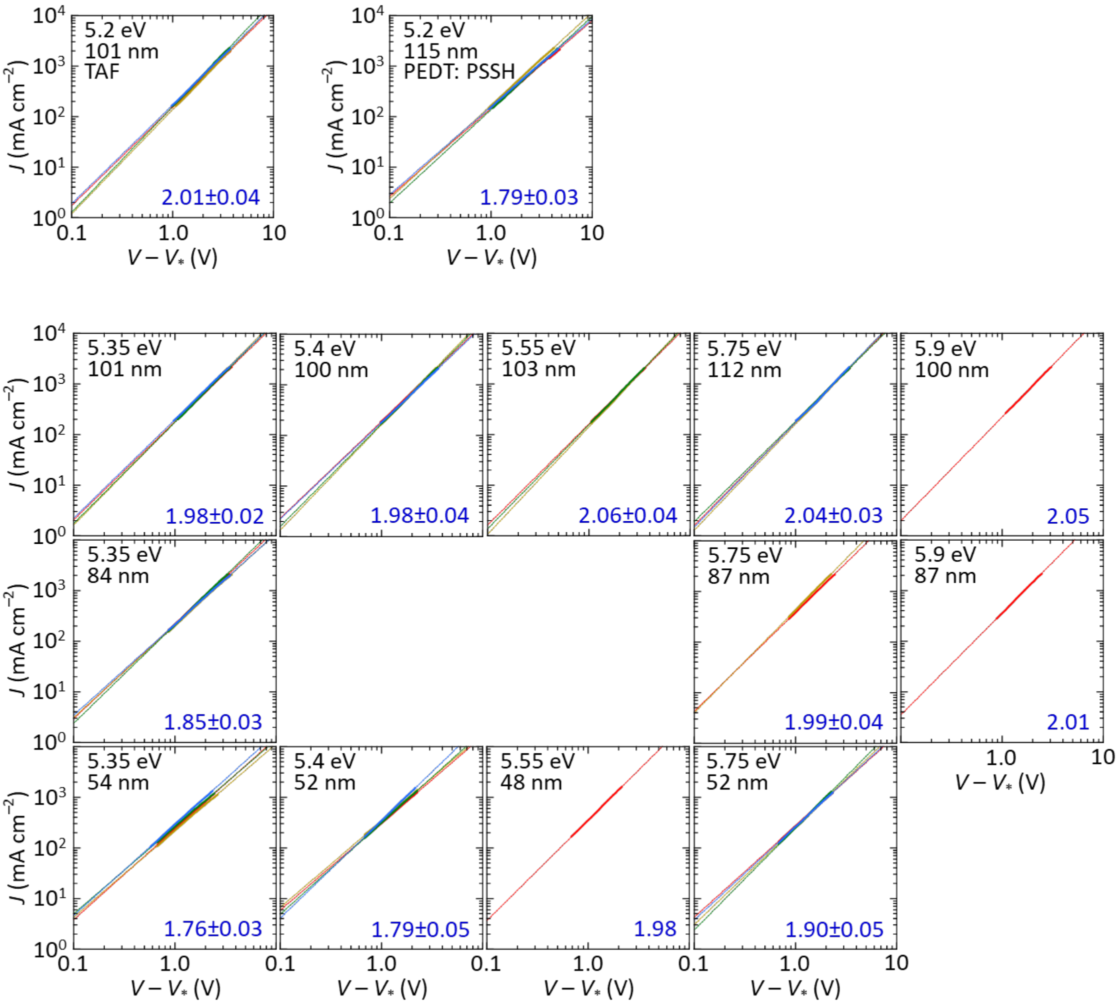

Supplementary Figure 11. *JV* characteristics of diodes for evaluation of the Mott–Gurney index *m*. Data from Supplementary Figures 3 and 10. The index is defined as: $m = \frac{d\log J}{d\log(V-V_*)}$. The value of m is given at bottom right of each plot, together with its standard error, wherever applicable. The R^2 correlation coefficient is better than 0.999 in all cases. The ideal m value of 2 is found for thicker films, and for diodes with better hole injection contacts, as expected. Simulation with carrier-density-dependent mobility of the form given in Table 1 confirms that m indeed adopts a value of 2.0 for Ohmic contacts over the current–voltage range here.

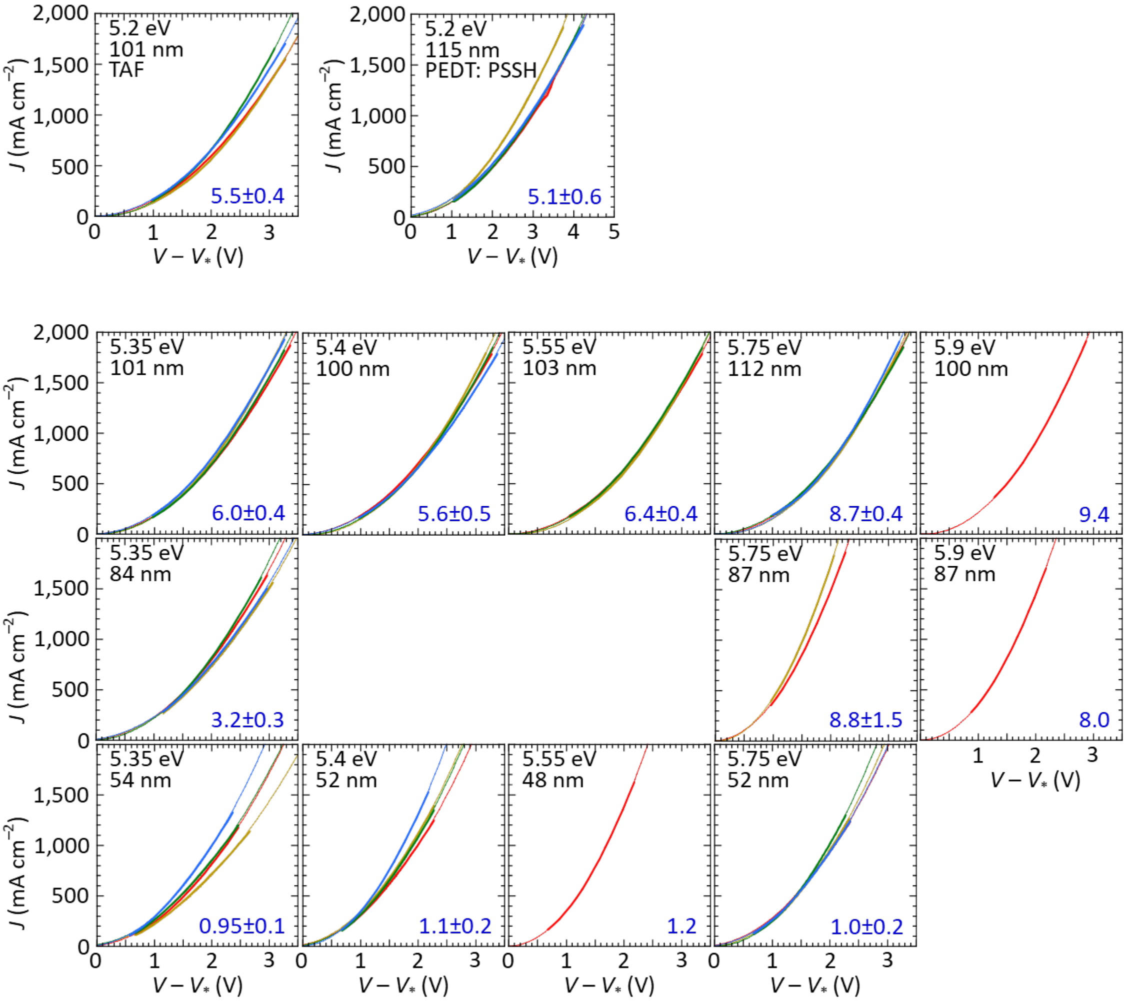

Supplementary Figure 12. *JV* characteristics of diodes fitted to the ideal Mott–Gurney equation. Data from Supplementary Figures 3 and 10. The value of μ_{eff} from $J = \frac{9}{8}\varepsilon_r\varepsilon_o\mu_{eff}\frac{(V-V_*)^2}{d^3}$ is given at bottom right of each plot, in units of 10^{-4} cm^2 V^{-1} s^{-1}, together with its standard error, wherever applicable. The V_* values are as given in Supplementary Figure 10. Dielectric constant ε_r is assumed to be 3.0. The R^2 correlation coefficient is better than 0.999 in all cases.

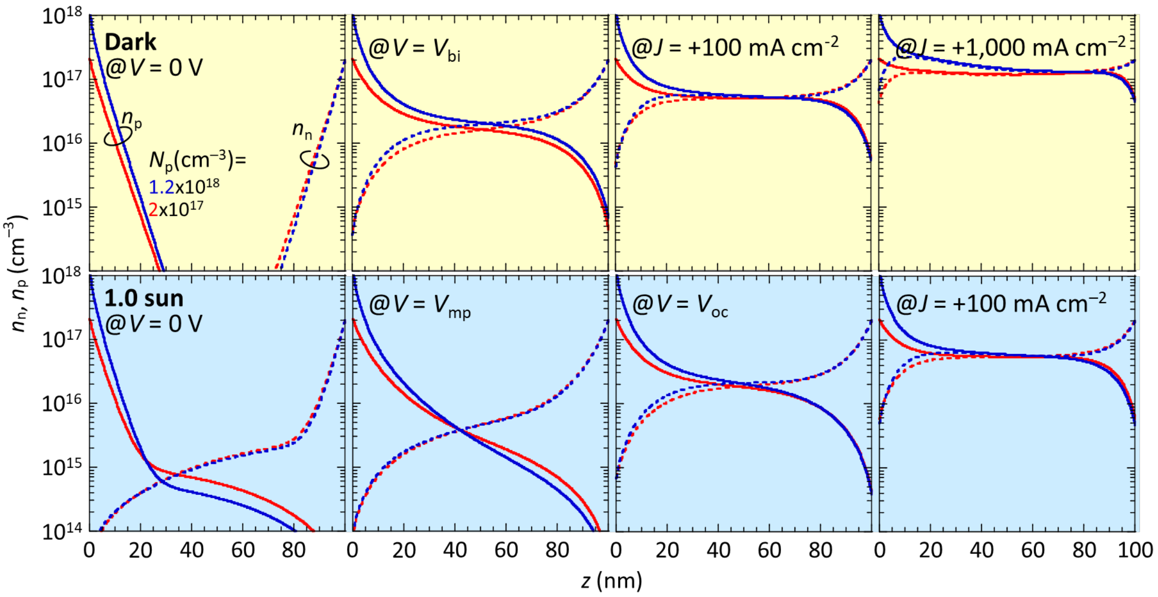

Supplementary Figure 13. Computed distance-dependent carrier density profiles at different conditions. Legend: hole density n_p (solid lines); electron density n_n (dotted lines), for hole density at hole contact N_p of 2 x 10^{17} (red) and 1.2 x 10^{18} cm^{-3} (blue), corresponding to $\phi = \phi_{pin} + 0.1$ eV, and $\phi_{pin} + 0.8$ eV, respectively. V_{mp} is the maximum power voltage. Methodology: Drift–diffusion–generation with bimolecular recombination and constant carrier mobilities for 100-nm-thick cells. Electron density at electron contact N_n is taken to be 2 x 10^{17} cm^{-3}. Other parameters are given in Table 1 of the main article. Minority carrier density at the injection/collection contact depends on contact selectivity, but does not significantly alter the computed *JV* characteristics nor majority carrier density profiles.

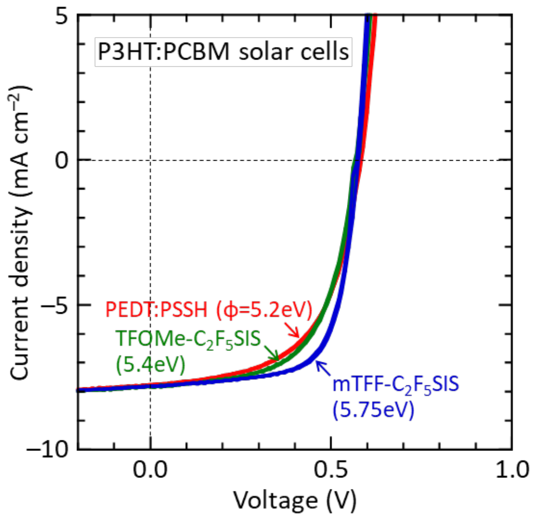

Supplementary Figure 14. Representative solar cell characteristics of P3HT:PCBM solar cells with different HCL work functions. Cell structure: ITO/30-nm HCL/80-nm P3HT:PCBM (1:0.8 w/w)/30-nm Ca/Al, where the P3HT:PCBM layer was annealed at 80°C for 10 min on hotplate in nitrogen glovebox before Ca deposition. Measurement conditions: AM1.5G (simulated) at 100 mW cm^{-2}. Typical parameters: V_{oc} = 0.59 ± 0.01 V, J_{sc} = 7.9 ± 0.02 mA cm^{-2}. However, FF increases from 0.57 ± 0.01 for PEDT:PSSH as HCL (reference, same as literature), to 0.61 ± 0.01 for TAF with ϕ = 5.4 eV, and 0.70 ± 0.01 for TAF with ϕ = 5.75 eV. Such high FF is unprecedented for the conditions used. References: W. Ma, C. Yang, X. Gong, K. Lee, A.J. Heeger, Adv. Funct. Mater. 15 (2005) 1617; V.D. Mihailetchi, H.X. Xie, B. de Boer, L.A. Koster, P.W.M. Blom, Adv. Funct. Mater. 16 (2006) 699; J.K. Tan, R.Q. Png, C. Zhao, P.K.H. Ho, Nature Comm, 9 (2008) 3269.

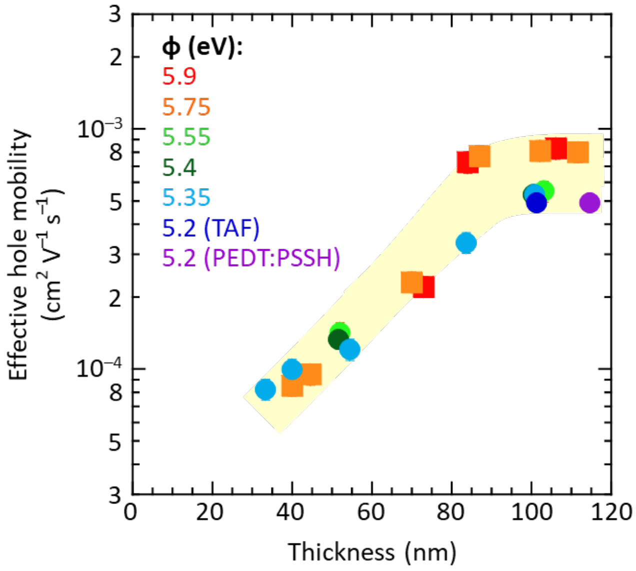

Supplementary Figure 15. Effective hole mobility of PBDTTPD:PCBM films as a function of film thickness, on different HILs. Device structure: ITO/HIL/PBDTTPD:PCBM/Ag, where HIL is hole injection layer from the family of TAFs or PEDT:PSSH. Methodology: *JV* fitting to Mott–Gurney equation**.** Data symbols are color-coded by ϕ of HIL. Yellow highlight is a guide-to-the-eye. For film thicknesses larger than *ca*. 80 nm, a higher ϕ induces a higher hole mobility, but this effect disappears at small film thicknesses, where hole mobility appears to decrease exponentially. Reason is unknown, but tentatively attributed to change in polymer morphology in the very thin films, as indicated by optical spectroscopy. Film thickness uncertainty, ± 5 nm.

**HCL**	**ϕ (eV)**	**V_{oc} (V)**	**J_{sc} (mA cm^{-2})**	**FF**	**$R_{s,oc}$ (Ω cm^2)**	**PCE (%)**
PEDT:PSSH	5.2	0.923 ± 0.001	9.25 ± 0.1	0.550 ± 0.006	22.0 ± 0.5	4.8 ± 0.1
TFOMe-CF_3SIS	5.2	0.895 ± 0.004	8.9 ± 0.05	0.640 ± 0.003	10.7 ± 0.5	5.11 ± 0.05
TFB-CF_3SIS	5.35	0.929 ± 0.001	8.85 ± 0.05	0.667 ± 0.003	6.4 ± 0.3	5.47 ± 0.05
TFOMe-C_2F_5SIS	5.4	0.916 ± 0.001	8.85 ± 0.05	0.680 ± 0.003	5.4 ± 0.2	5.52 ± 0.05
TFB-C_2F_5SIS	5.55	0.931 ± 0.001	8.9 ± 0.05	0.692 ± 0.003	5.7 ± 0.2	5.72 ± 0.05
mTFF-C_2F_5SIS	5.75	0.959 ± 0.001	9.05 ± 0.05	0.716 ± 0.003	5.9 ± 0.2	6.21 ± 0.05
pTFF-C_2F_5SIS	5.9	0.963 ± 0.001	9.05 ± 0.05	0.706 ± 0.006	6.0 ± 0.3	6.1 ± 0.1

Supplementary Table 1. Summary of cell parameters of ITO/20-nm HCL/100-nm PBDTTPD:PCBM (1:1.5 w/w)/30-nm Ca/Al cells, measured under simulated AM1.5G irradiance of 100mW cm^{-2}, spectral-mismatch corrected, 298 K.
